# StRAP: An Integrated Resource for Profiling High-Throughput Cancer Genomic Data from Stress Response Studies

**DOI:** 10.1371/journal.pone.0051693

**Published:** 2012-12-17

**Authors:** Seth Johnson, Biju Issac, Shuping Zhao, Mohit Bisht, Orieta Celiku, Philip Tofilon, Kevin Camphausen, Uma Shankavaram

**Affiliations:** 1 Radiation Oncology Branch, National Cancer Institute, National Institutes of Health, Bethesda, Maryland, United States of America; 2 SAIC-Frederick, Inc., National Cancer Institute-Frederick, Frederick, Maryland, United States of America; 3 Division of Bioinformatics, Sylvester Comprehensive Cancer Center, University of Miami, Florida, United States of America; Institute for Systems Biology, United States of America

## Abstract

The increasing availability and maturity of DNA microarray technology has led to an explosion of cancer profiling studies for identifying cancer biomarkers, and predicting treatment response. Uncovering complex relationships, however, remains the most challenging task as it requires compiling and efficiently querying data from various sources. Here, we describe the Stress Response Array Profiler (StRAP), an open-source, web-based resource for storage, profiling, visualization, and sharing of cancer genomic data. StRAP houses multi-cancer microarray data with major emphasis on radiotherapy studies, and takes a systems biology approach towards the integration, comparison, and cross-validation of multiple cancer profiling studies. The database is a comprehensive platform for comparative analysis of gene expression data. For effective use of arrays, we provide user-friendly and interactive visualization tools that can display the data and query results. StRAP is web-based, platform-independent, and freely accessible at http://strap.nci.nih.gov/.

## Introduction

DNA microarrays are successfully being used to classify tumors and identify novel biomarkers associated with cancer (for some recent reviews see [1]). Genetic variants and differences in personal genomes not only impact cancer profiles but are often responsible for how the patient and the cancer respond to treatment. In particular, the response to cellular stress, whether induced by cytotoxic drugs, hypoxia, or ionizing radiation can vary greatly, and its genetic basis is subject of much interest. We are especially interested in elucidating the genetic basis of radiotherapy response in search of highly-predictive genetic signatures. Radiotherapy is a core component of cancer treatment [2] but has been relatively under-studied: a glimpse at public resources like Pubmed or array databases shows that radiotherapy studies constitute less than 1% of the total number of records.

Typically, each individual study involves a number of statistical and quantitative analysis steps (see [3] for a summary of typical steps), and can point to gene and gene products that are crucial for disease and treatment. However, the sparse, high-dimensional nature of the microarray data space [4], and the large number of genes involved in often subtle and complex pathways, necessitate meta analyses for comparing and aggregating results from different studies. Cross-platform compatibility can only be achieved once within-platform consistency issues have been fully addressed and the results of such studies are as good as the gene identification method. MAQC consortium has generally found that proper sample preparation is sufficient to dramatically enhance multilab and multiplatform correlations [5]. The utility of such analyses was documented in the implementation of the CellMiner tool, a web based program for the integration of molecular profiling data at DNA, RNA, protein, and pharmacological levels on the widely studied NCI-60 cancer cells [6]. Several other studies found added complexity for meta analysis due to considerable diversity in source, sample, and platform types [7]–[9]. The two major technologies of microarrays differ in the basic design, cDNA microarrays use full-length transcripts printed onto the slides and oligonucleotide based arrays constitute a shorter- oligonucleotides synthesized in situ. A major design question is whether to measure the expression levels from each sample on a different microarray (using single-color, or single-channel, arrays), or instead to compare relative expression levels between a pair of samples on each microarray (two-color or two-channel arrays). There are tradeoffs between the two approaches. Single-color arrays allow for more flexibility in analysis, while two-color arrays can control for some technical issues by allowing a direct comparison in a single hybridization [10]. A recent comparison of single- and two-color methods on the same platforms found good overall agreement in the data produced by the two methods [11]. The Z score transformation procedure for normalizing data is a familiar statistical method in both neuroimaging and psychological studies and recently been used in the meta analysis of microarray datasets from different platforms [12], and is especially suited for database development [13].

The wealth of data has also brought about the creation of a wide range of resources. On one end of the spectrum, data repositories like Gene Expression Omnibus (GEO) [14] provide access to raw experimental data; on the other end, tools like ONCOMINE [15] more ambitiously, but typically at a cost, provide facilities for meta analysis of array data. However, to our knowledge, none of the existing free resources focus on stress response or radiotherapy studies combined with visualization outputs.

We develop StRAP, a free web-accessible resource to address the need to query, compare, profile, and visualize results from different microarray experiments. StRAP hosts data from diverse cancer studies (currently from 12 different tissue types), and will be further extended in the future. We used Z scoring method to standardize data, since the internally normalized values do not change with subsequent addition of new datasets. All data are mapped to Entrez Gene identifiers for consistency in comparison. The user-friendly interface facilitates exploration by a wide-range of researchers, including those with little expertise in bioinformatics.

In the remainder of this paper we briefly describe StRAP’s construction and core features.

## Materials and Methods

### Architecture

**Figure 1:**
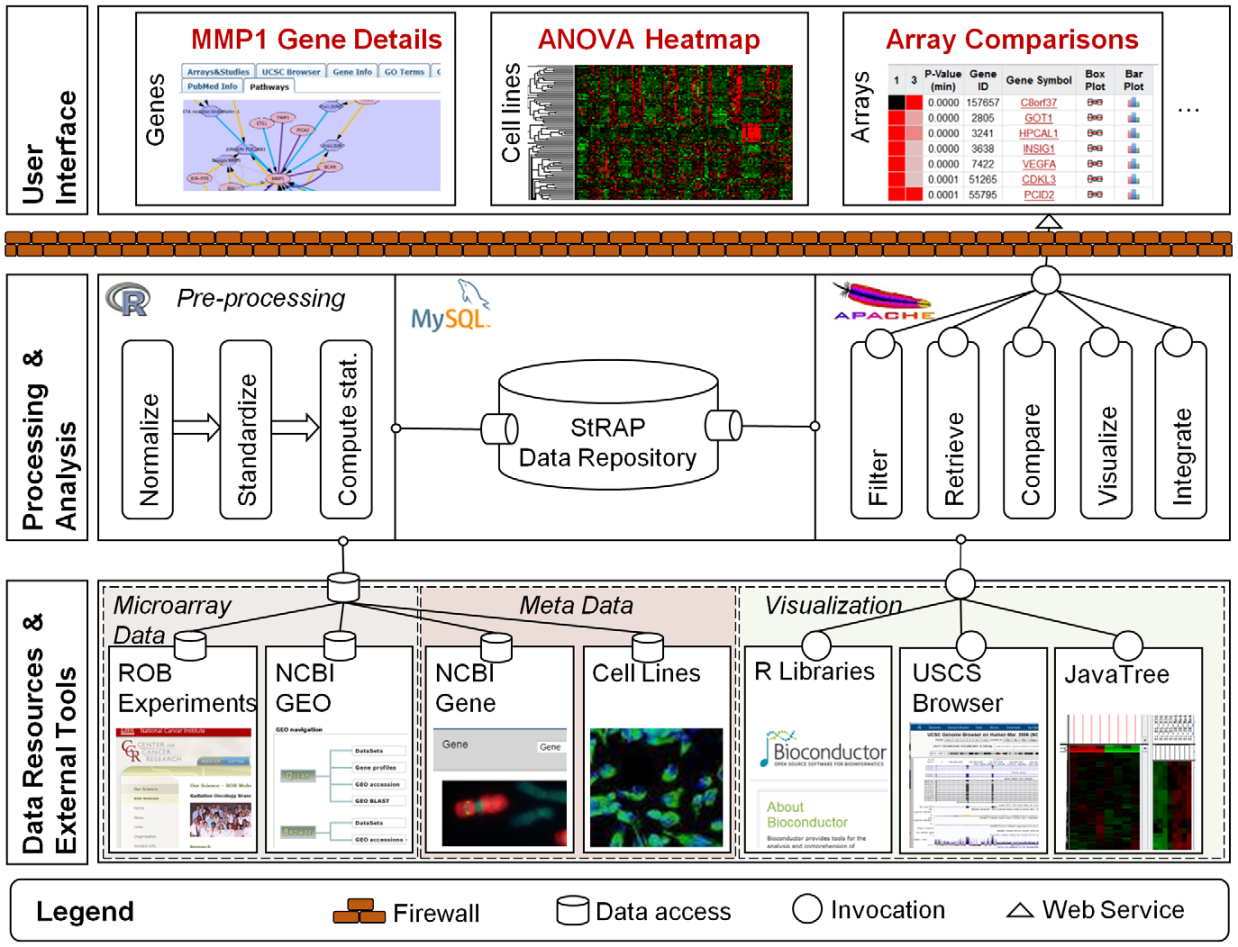
StRAP architecture diagram. The diagram represents a runtime view of the architecture of StRAP. The lower tier represents the sources of experimental data, meta data, and external tools that are invoked to visualize the data. The middle tier represents how the data are processed, stored, and made available to the user. The right hand side of the middle tier represents the analysis “services” that are available at runtime to the user. The higher tier represents the user interface, and is organized around three main modules (Genes, Cell lines, and Arrays).

The runtime architecture of StRAP is described in Figure 1. The architecture is 3-tiered. The basic design of the architecture is an enhancement of our previously published CellMiner tool [6]. The bottom tier represents the sources of experimental (microarray), meta (cell line) data, and external tools that are invoked to visualize the data. The middle tier represents how the data are processed, stored, and made available to the user. The pre-processing steps were performed before deployment. At this stage, data from the lower tier were accessed, processed (using R scripting), and stored in the StRAP data repository (comprised of a MySQL database, and other files stored on the server file system). The right hand side of the middle tier represents the analysis “services” that are available at runtime to the user. These include filtering of data (according to user constructed queries), visualization of results, and the options to download the data. These services are made available as web-services and are hosted on an Apache server. The top tier represents the user interface (implemented using PHP, Javascript, AJAX, and HTML), and is organized around three main modules (Genes, Cell lines, and Arrays).

### Data Repositories

Four main data repositories reside at the backend of StRAP: (1) Gene associated annotation information derived from the National Center for Biotechnology Information (NCBI, http://www.ncbi.nlm.nih.gov/), (2) Pre-processed gene expression microarray molecular profile data (including pre-computed statistics), (3) Metadata on cell lines, and (4) Metadata on platform-associated information.

The structured layout of the tables promotes efficient querying and integration of phenotypic data, metadata and molecular profile information from various studies. The database supports multiple concurrent query sessions.

The repositories are stored as a MySQL relational database (http://www.mysql.com).

### Data Preparation

The microarray data were obtained as raw files whenever available or else as author deposited normalized files from the GEO database [14], ArrayExpress [16], or in-house experiments. Two platform types are predominantly used in these studies: cDNA two-color (National Cancer Institute- ROSP 8K Human Array and Agilent whole human genome microarrays), and single color arrays (currently we house Affymetrix and Illumina gene chip data).

The raw data were assessed for quality and normalized by the Lowess [17], or MAS5 [18]methods for cDNA, and Affymetrix arrays, respectively. Z-score transformation was used to obtain a uniform scale across different studies and platforms, which is necessary for comparing data from different studies. Pre-computed statistical tests were performed at three nested-level complexity.

- At the top level, each study is subjected to ANOVA analysis performed between all controls and cases to give an overall significance of the study design.
- A tissue level ANOVA analysis is implemented as a second tier of comparison between all the controls and cases for each tissue type in a study.
- At the experiment level, for each cell-line/sample, a case-control comparison is performed by t-test analysis.

Pre-processing and computation of statistical tests are performed in the R environment (http://www.r-project.org/).

### Interface

The front end interface is a web-based application implemented using R, PHP (http://www.php.net/) and Python (http://www.python.org/). The application is deployed on an Apache HTTP server (http://httpd.apache.org/) at the National Cancer Institute (NCI).

### Core Features

Data access and presentation is organized around three main concepts or modules: (1) Genes, (2) Cell lines, and (3) Arrays. Flexible user-defined data queries can be initiated from any of the modules; the data visualization options for the results are displayed in integrated views and may, depending on the query, involve cross-talk between modules. Several links to external resources promote a systems biology approach. Table 1 gives a summary of core features for each module. Pre-computed statistics (as described in the previous section) enable display of efficient and intuitive graphs.

**Table 1:** StRAP modules functionality.

	Gene module			Cell line module		Array module		
Description	Gene level comparison of one or more studies queried by gene related keyword search, identifier, or GO terms.		Gene identifier conversion utility	Cell line based comparison of one or more studiesselected individually, by tissue type, or manuallyfrom a list of all available studies		Overview of all studies, or studies by tissue. Enables comparison of studies by common cell lines, or union of genes. Enables downloading of data.		
Results	List of associated studies	Gene information	Mapping of gene identifiers of one typeto another	List of selected studies	Cell line information	Option tocompare studiesby cell line	Option tocompare studiesby gene	Study information
Details of meta information		UCSC genome browser, Pubmed references, GO terms, and Pathway Commons networks	Table of mapped gene identifiers		Description of origin andsource, experimental details,and source referenceinformation			Experimental details, study reference information, contributors, and data download
Visualization of multi-gene query. A multi-study comparison by Metamap, or a single or multi-study comparison by Heatmap	Yes			Yes		Yes	Yes	
Visualization of Single gene query. A single or multi-study comparison at study level by Boxplot, and at Experiment level by Barplot	Yes			Yes		Yes	Yes	

### Genes

The genes module enables gene-centric queries of the StRAP microarray studies. Queries can be based on gene or protein identifiers, synonyms, gene descriptions, or chromosome location. The results include associated arrays and studies, and a compilation of gene-annotation information, spatial localization within the genome visualized in the UCSC Genome browser [19], and network neighborhood maps generated from protein-protein interaction networks [20]. Queries can also be constructed using gene lists defined by the user or generated, for example, from Gene Ontology (GO) terms [21].

**Figure 2:**
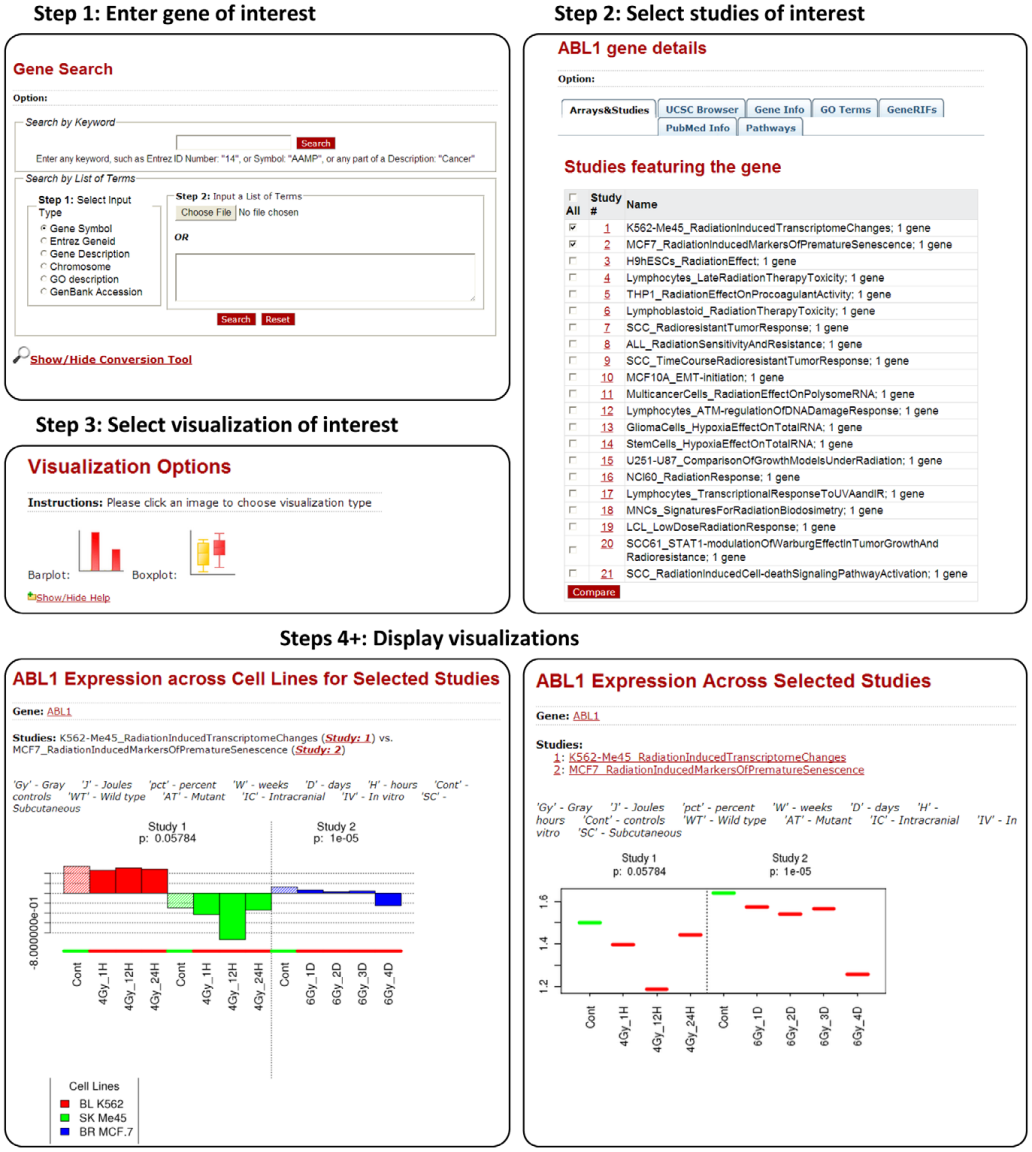
Example of a workflow initiated from the Genes module. Typically, a workflow initiated from the Genes module involves 1) entering a gene of interest (or list of genes), 2) displaying and selecting studies featuring the gene, 3) choosing a visualization option, and 4+) displaying and inspecting the chosen visualization. The example shown is for gene “ABL1.”.

A typical gene-centric query (see Figure 2 for an example workflow) starts by identifying studies profiling the expression of a gene (list) of interest. The expression profiles and their statistical significance are then visualized via boxplots, and barplots (showing study-level, and experimental-level case-control differences). If the input involves a list of genes, an interactive heatmap option enables viewing expressions of genes in selected studies. The heatmap is visualized using the Java Treeview program [22].

As an added convenience, the genes module includes a gene identifier conversion utility, which can be used to map from one type of gene identifier (for example, Entrez gene symbol) to another (for example, Entrez geneid).

### Cell Lines

**Figure 3:**
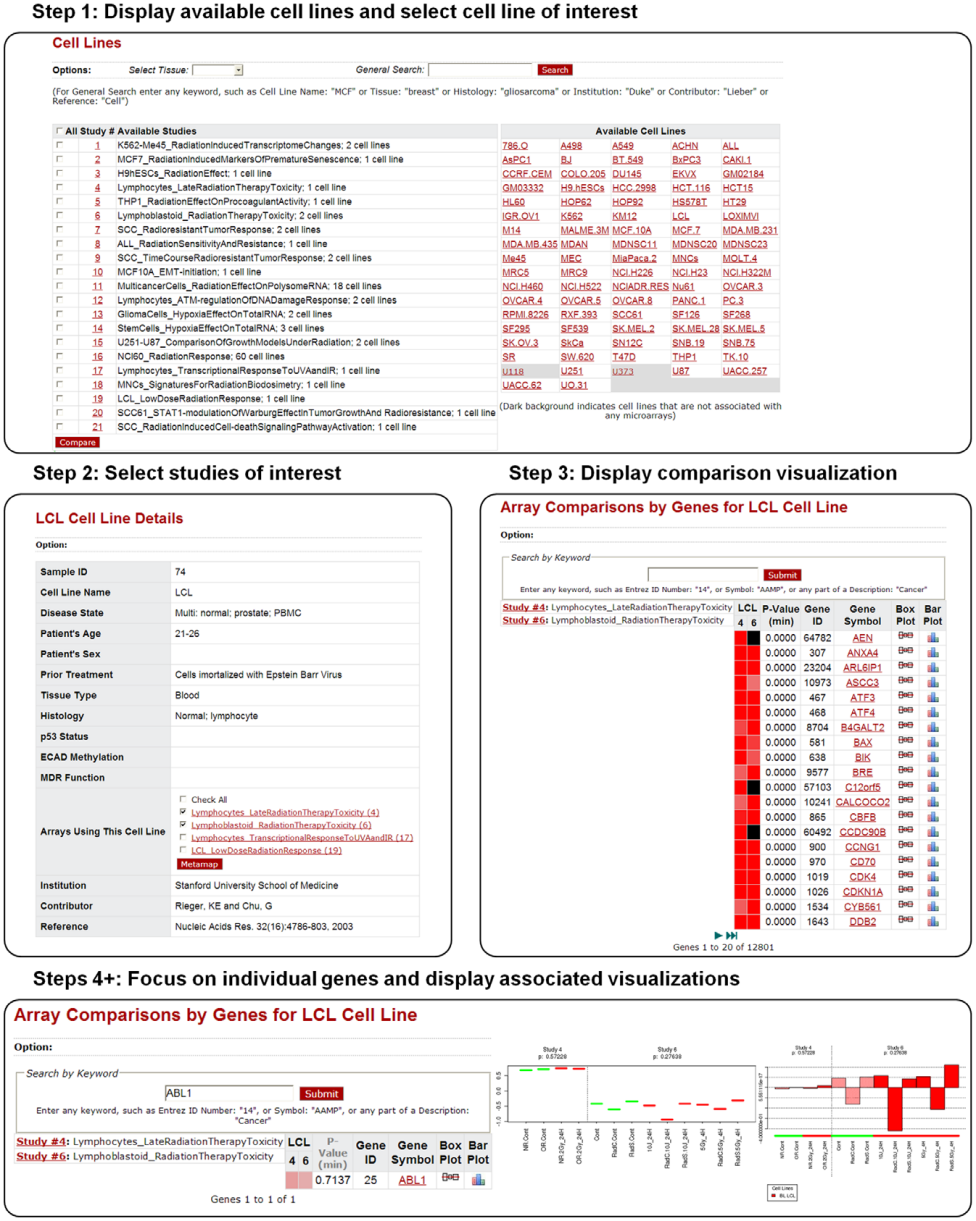
Example of a workflow initiated from the Cell lines module. The Cell lines initiated workflow typically starts with 1) selection of a cell line (or tissue) of interest (here “LCL”), 2) inspection of the cell line metadata, and associated studies, 3) comparison of studies of interest with a metamap showing significance of differential expression of individual genes for the given cell line, and 4+) inspecting individual genes via barplots and boxplots.

The cell lines module provides metadata on available cell lines and associated studies. Queries in this module are tailored to allow selection of complete studies, by tissue of origin, or individual cell line. Comparisons can be made for samples within a study or across studies. (See Figure 3 for an example workflow.) Differentially expressed genes in studies of interest are identified based on case-control t-test analyses (cell line selection) and ANOVA analysis (studies with more than one group). The default filter is set to p≤0.05, but can be customized by the user.

### Arrays

The arrays module provides an overview of the current contents of the database, including the number of studies, information on platforms, contributors, and available meta-information. Pre-processed data or data from the original source can be downloaded from this module. Integrated queries from this module allow performing comparison of studies by common samples or union of genes within the selected studies.

**Figure 4:**
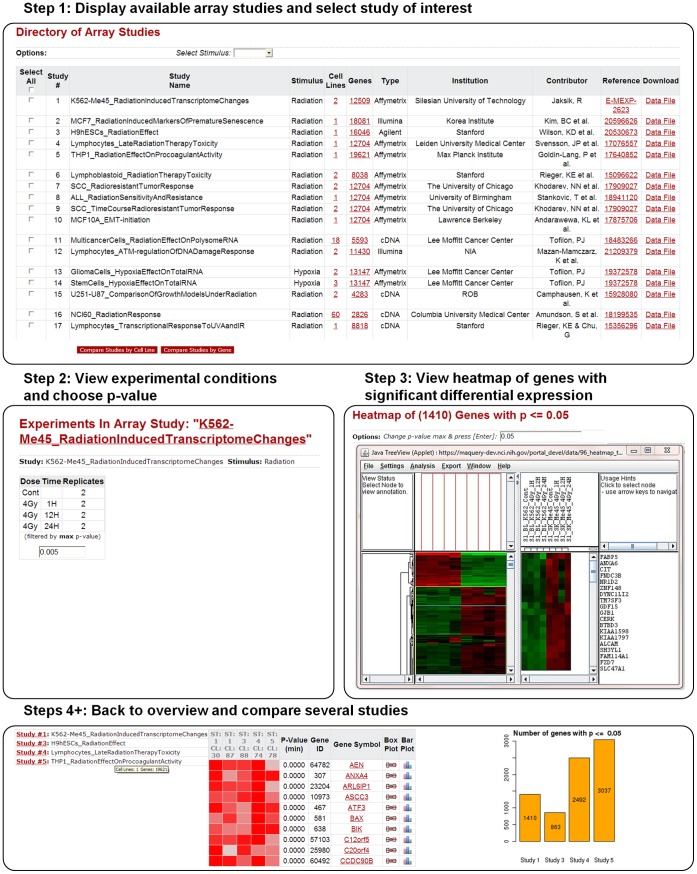
Example of a workflow initiated from the Arrays module. The Arrays workflow typically starts with 1) inspection of available arrays and selection of a study of interest, 2) viewing of experimental conditions and selection of a p-value threshold for significance of gene expression differentiation, and 3) study of expressions heatmap. Comparison of several arrays can also be initiated from the overview page.

An example workflow is shown in Figure 4. Arrays can be filtered by the select stimulus used in the study. Given our interest in effects of ionizing radiation, most of the arrays in the repository have “radiation” as stimulus.

**Figure 5:**
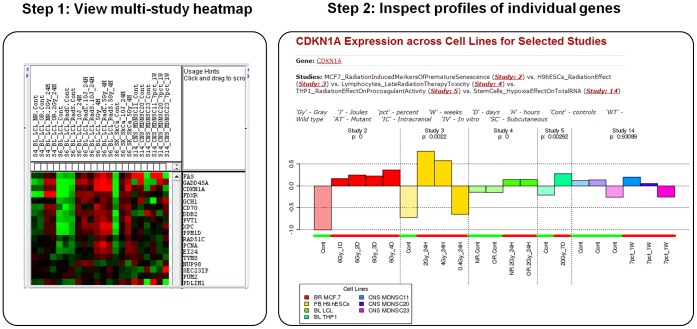
StRAP validation. Differential expression of twenty-four genes identified by Rieger and colleagues [24] to be important for radiation response. In Step 1 shows a multi-study heatmap (for Studies 4, 6, and 14). In Step 2 CDKN1A gene profile was compared in studies with (Studies 2–5), and without radiation as stimulus (Study 14 with hypoxia response).

### Validation

Radiation therapy is a core component of cancer treatment. However, radiation response often varies considerably among different patients [23]. Therefore, it is important to identify genes predictive of radiation response. Equally important is to validate the results of an analysis in independent data with similar experimental design.

To illustrate the functionality of StRAP, we used a study by Rieger and colleagues [24] on peripheral blood lymphoblastoid cells derived from patients with acute radiation toxicity and control group of patients with mild toxicity. Using gene expression profiling, the authors reported 24 highly predictive genes of radiation response. We sought to explore the expression of these 24 genes in several independent studies from StRAP database, and found 18 genes significantly changed among the selected studies. To test if we can reproduce the authors findings, we first selected 3 studies, 2 studies (studies 4 and 6) containing lymphoblastoid cells treated with different doses of radiation, and as a negative control, we chose 1 study (Study 14) with stem cells from CNS tissue with hypoxia stimulus. A multi-study heatmap (Figure 5, Step 1) on the gene subset showed a selective up regulation of the gene subset in studies 4 and 6 but, not in study 14, confirming the role of these genes in response to radiation. Of particular, CDKN1A is a DNA damage response, cell cycle regulating gene reported to be induced by radiation [25], [26]. We explored the comparative profiling of CDKN1A gene in a range of studies with diverse cell lines from our database that are treated with (Studies 2–5) or without radiation as stimulus (Study 14). A comparative gene profiling across multiple studies (Figure 5, Step 2) showed a significant induction of the gene selectively in radiation treated studies. In addition the induction is found to have no effect at low dose radiation (0.4 Gy in Study 3) indicating cellular response to radiation is dependent on dose rate used.

## Conclusions

StRAP is an open-access resource developed primarily to support research on the effects of stress with major emphasis on ionizing radiation on cancer in a systems-biology context. Currently data from twenty one studies have been integrated and made accessible through extensive query options, and a user-friendly web-based interface. Supported by statistical and quantitative analysis methods in the background, the resource overcomes the limits of databases dedicated to raw data exploration, making it possible to infer nontrivial knowledge (such as the differentially expressed genes in multiple studies).

Currently because of the limitation of the number of studies available, it may have limited biological significance. However, the framework of the database is flexible and would allow extensions with data from other types of cancer studies that will help in novel findings.

The database will be periodically updated with new studies and features. We plan, for example, to enable construction of interaction networks using literature text-mining, and information from the Human Protein Reference Database (HPRD) [27] and gene set enrichment analyses and visualizations.
